# A role for lakes in revealing the nature of animal movement using high dimensional telemetry systems

**DOI:** 10.1186/s40462-021-00244-y

**Published:** 2021-07-28

**Authors:** Robert J. Lennox, Samuel Westrelin, Allan T. Souza, Marek Šmejkal, Milan Říha, Marie Prchalová, Ran Nathan, Barbara Koeck, Shaun Killen, Ivan Jarić, Karl Gjelland, Jack Hollins, Gustav Hellstrom, Henry Hansen, Steven J. Cooke, David Boukal, Jill L. Brooks, Tomas Brodin, Henrik Baktoft, Timo Adam, Robert Arlinghaus

**Affiliations:** 1 Laboratory for Freshwater Ecology and Inland Fisheries (LFI) at NORCE Norwegian Research Centre, Nygårdsporten 112, 5008 Bergen, Norway; 2 INRAE, Aix Marseille Univ, Pôle R&D ECLA, RECOVER, 3275 Route de Cézanne - CS 40061, 13182 Cedex 5 Aix-en-Provence, France; 3 Institute of Hydrobiology, Biology Centre of the Czech Academy of Sciences, České Budějovice, Czech Republic; 4 Movement Ecology Lab, Department of Ecology, Evolution, and Behavior, Alexander Silberman Institute of Life Sciences, The Hebrew University of Jerusalem, 102 Berman Bldg, Edmond J. Safra Campus at Givat Ram, 91904 Jerusalem, Israel; 5 Institute of Biodiversity, Animal Health and Comparative Medicine, College of Medical, Veterinary and Life Sciences, University of Glasgow, Graham Kerr Building, Glasgow, G12 8QQ UK; 6 Faculty of Science, Department of Ecosystem Biology, University of South Bohemia, České Budějovice, Czech Republic; 7 Norwegian Institute of Nature Research, Tromsø, Norway; 8 University of Windsor, Windsor, ON Canada; 9 Department of Wildlife, Fish, and Environmental Studies, Swedish University of Agricultural Sciences, Umeå, Sweden; 10 Karlstads University, Universitetsgatan 2, 651 88, Karlstad, Sweden; 11 Fish Ecology and Conservation Physiology Laboratory, Department of Biology, Carleton University, Ottawa, ON Canada; 12 Institute of Entomology, Biology Centre of the Czech Academy of Sciences, České Budějovice, Czech Republic; 13 Technical University of Denmark, Vejlsøvej 39, Building Silkeborg-039, 8600 Silkeborg, Denmark; 14 Bielefeld University, Universitätsstraße 25, 33615 Bielefeld, Germany; 15 Department of Biology and Ecology of Fishes, Leibniz Institute of Freshwater Ecology and Inland Fisheries, Bergen, Germany; 16 Division of Integrative Fisheries Management, Humboldt-Universität zu Berlin, Bergen, Germany

**Keywords:** Telemetry, Sensor, Biologging, Movement ecology, Fish ecology

## Abstract

Movement ecology is increasingly relying on experimental approaches and hypothesis testing to reveal how, when, where, why, and which animals move. Movement of megafauna is inherently interesting but many of the fundamental questions of movement ecology can be efficiently tested in study systems with high degrees of control. Lakes can be seen as microcosms for studying ecological processes and the use of high-resolution positioning systems to triangulate exact coordinates of fish, along with sensors that relay information about depth, temperature, acceleration, predation, and more, can be used to answer some of movement ecology’s most pressing questions. We describe how key questions in animal movement have been approached and how experiments can be designed to gather information about movement processes to answer questions about the physiological, genetic, and environmental drivers of movement using lakes. We submit that whole lake telemetry studies have a key role to play not only in movement ecology but more broadly in biology as key scientific arenas for knowledge advancement. New hardware for tracking aquatic animals and statistical tools for understanding the processes underlying detection data will continue to advance the potential for revealing the paradigms that govern movement and biological phenomena not just within lakes but in other realms spanning lands and oceans.

## Introduction

Animals are born, they move and reproduce, and then they die. This simple model of life supports all ecological processes and movement has therefore emerged as a frontier for animal research [131, 145, 200]. Movement ecology is a multiscale branch of ecology operating from cells to whole animals, populations, and communities across short or long distances for brief intervals or even spanning generations. Where do animals move, when, why, and how? These are foundational ecological questions and the answers have significant implications for our understanding of the natural world and the management of resources that we depend upon [172, 200, 210].

Significant and rapid advances have been made in our understanding of movement ecology coincident with the introduction and proliferation of electronic tags to remotely measure animal behaviour and physiology [131, 145]. The capacity to simultaneously monitor movement and the environment yields great opportunity but also significant responsibility to identify focal systems with which to make inferences [117]. To this end, Hays et al. [117] presented a list of research priorities related to megafaunal movement, specific to a system where research is inherently challenging and limited by the vast scale of latitudinal and longitudinal connectivity coupled with profound depths: the marine environment. This daring focus renders many studies, particularly those that concentrate on community scales and consider interactions among species, logistically challenging.

Lakes are ideal study systems for testing ecological paradigms, including for movement ecology. For over a century, lakes have been acknowledged for providing ample opportunities to investigate ecological, behavioral and evolutionary questions at manageable scales [86]. Lakes are highly important venues for studying ecology because freshwater habitats are among Earth’s most valuable, rare, and threatened ecosystems [79, 240]. As relatively closed ecosystems with less influence from distant processes [192], animal movement can be linked more directly to local phenomena, including weather patterns and the immediate ecological community. Lakes offer a great diversity of structural and physical processes with similarity at local scales but substantial variation in fish assemblages and aquatic communities across latitudes and longitudes. Small lakes can effectively be covered by an array of acoustic receivers in a comparable design to a bay or coastal area in the ocean or a great lake but with higher resolution of the processes operating within. Replication of studies in multiple lakes offers the potential for robust inferences from ecological and manipulative experiments [50, 255], including how environmental stressors and ecological interactions modify movement behaviour. For these reasons, lakes have long provided essential venues for ecological inquiry and many paradigms have emerged from the flexibility, observability, and replicability of research in lakes, including ecological regime shifts [253], predation risk effects [304], predator-prey-habitat complexity relationships [95], trophic positioning from stable isotopes [293], habitat degradation [256], and ecological speciation [258, 262].

## Lakes as venues for movement ecology research

We submit that lakes provide perfect venues in which to investigate many of the most fundamental questions of movement ecology with results that are scalable to larger systems. To that end, we turn to the key questions of marine megafaunal movement ecology presented by Hays et al. [117] and suggest that many of these questions can also be applied to whole lake studies. We interpret these questions as relevant across mobile taxa and not limited to the marine environment or to megafauna specifically. We posit that answering these questions will yield significant advances in our understanding of movement ecology independent of the system. Our approach is to draw on our experiences working in acoustically instrumented lake environments to discuss the vast opportunities these systems have to address 15 movement ecology questions identified by Hays et al. [117] that we agree will drive the movement ecology field forward in coming years. Each section is divided into three paragraphs in which we first describe key examples and potential connections, followed by questions using lakes as focal systems that could advance understanding, and finally the approaches that could accomplish this. We conclude this essay with a synthesis where we discuss the tools and approaches that we envision researchers applying to better understand the complexities of aquatic life for better habitat management, ecosystem conservation, and fundamental science.

### How can movement data be used to support conservation and management?

Aquatic biodiversity is in steep decline due to a range of anthropogenic factors, including habitat alterations [240]. There are also increasing examples of overfishing of freshwater stocks [232] and of other exploitation-induced issues [10, 167]. Movement data are key, yet underutilized to design effective conservation and management strategies, e.g., in the context of fisheries and conservation of freshwater fish and freshwater habitats [14, 71]. Lake tracking data can be used to identify seasonal and daily movements, dispersal, connectivity of habitats [115, 198], e.g., after stocking [193], behavioural diversification and its relation to individual fitness [150], capture probability [193], spawning site fidelity [149], stock boundaries among connected ecosystems and within ecosystems [67, 116], reactions to human influences, such as boat movement [135] or catch-and-release [15], and degree of fishing-induced mortality [120]. An obvious further application example from a conservation context is applying telemetry to examine the ability of freshwater protected areas to help heavily exploited fishes recover from heavy fishing pressure [236]. In this context, telemetry is useful to identify sites where encounters with fishing gears are rare.

Despite the opportunities, there are limited examples of fine-scale, whole-lake tracking studies that have realized the potential of informed management and conservation. The few systems that were or are in place have generated a number of highly relevant results. Baktoft et al. [15] used whole lake telemetry to assess the reactions of northern pike (*Esox lucius*) to handling, including catch-and-release. Jacobsen et al. [135] studied the response of different freshwater fish to boating, revealing limited impacts on the behaviour of freshwater fish. O’Connor et al. [214] showed that a one-time intensive stressor can have carry-over effects many months later during hypoxia in largemouth bass (*Micropterus salmoides*). Work in a small lake in Germany has revealed how angling can directly select on behavioural traits, such as habitat choice in perch (*Perca fluviatilis*) [194]. Similar research has been conducted in “lake-like” coastal systems where small-bodied coastal fish with limited home range were exposed to angling, revealing how angling could be a selective force on home range, activity, and chronotypes [7]. A ground baiting experiment at a whole lake scale showed how omnivorous fish respond to angler-induced bait and how this novel energy is embedded in certain trophic levels elevating secondary production [187]. Fine-scale acoustic telemetry has also been used to study restoration success in Toronto, Canada [297] and how exposure to pollutants affects the behaviour of Eurasian perch in the wild [148].

**Fig. 1:**
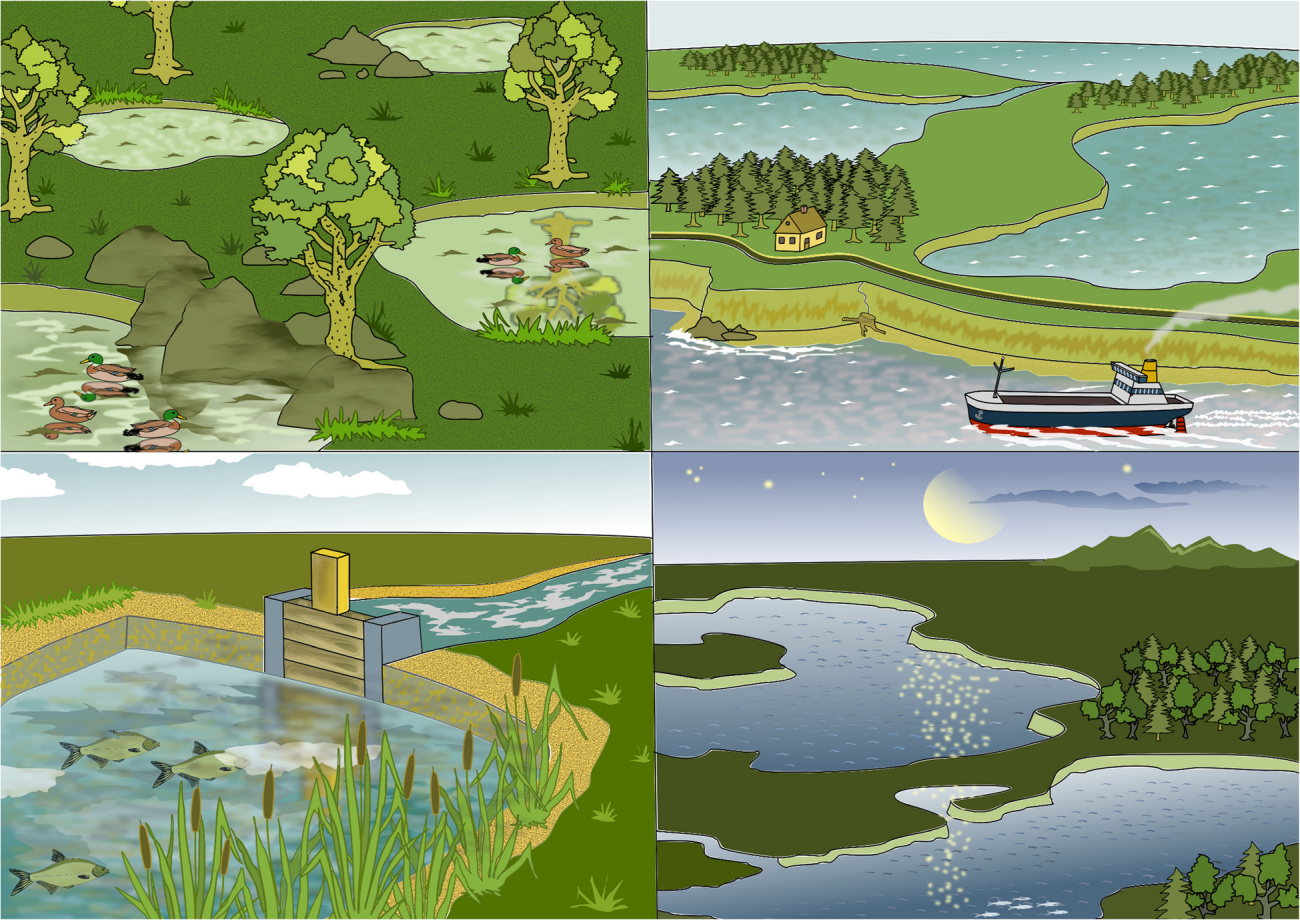
Lakes come in many shapes and sizes, all of which have the potential to be monitored using environmental sensors and telemetry to reveal the nature of animal movement. In this grid we show lake size scales between small (left half) and large (right half) and experiments can be conducted in isolation (a single lake, lower half) or in a replicated design (upper half). Finding matching lakes to replicate experiments allows a degree of control that is difficult or impossible to achieve in other systems. Moreover, scaling lakes from small to large allows a degree of environmental realism desired for the experiments, with animals in small lakes using all habitats but in large lakes habitat segregation and different competitive mechanisms emerging

**Fig. 2:**
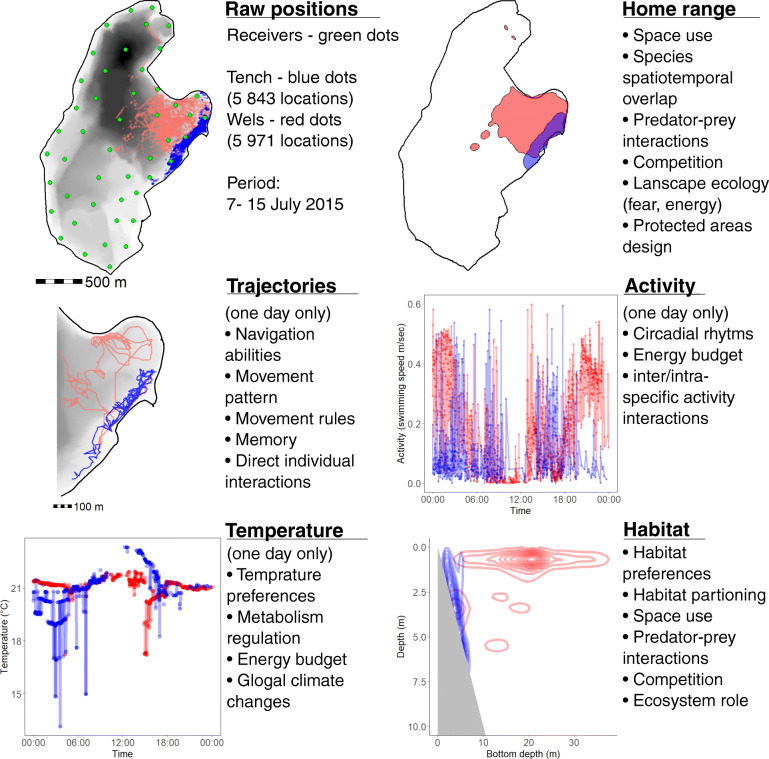
Acoustic telemetry yield data on the instrumented animal’s positions, path, space use, activity levels, temperature use, and habitat selection in up to four dimensions. Here, we illustrate how detections on a grid of acoustic receivers can be used to investigate patterns in the behaviour and physiology of free living fish to describe where, when, how, and why animals are moving. Together, lake telemetry studies are powerful tools for inquiry about processes and patterns in ecology

Compared to the oceans, spatially finite ecosystems such as ponds or lakes can offer replication and allow whole-ecosystem type experiments to be conducted with appropriate replicates (either in space or time) and with controls (e.g. manipulated vs. unmanipulated; Fig. 1). Before-after-control-impact studies are a gold-standard in the applied environmental sciences, particularly in freshwater ecology, and are particularly useful to identify how common conservation and management actions operate at ecologically realistic scales. Lakes offer excellent experimental arenas for such types of studies. Experiments could, for example, tackle questions of habitat enhancement or degradation, stocking and introductions, selective harvesting and effectiveness of protected areas. Smaller pond ecosystems could also be experimentally warmed to study impacts of climate change. Replicated lakes could be used to study impacts of invasive species, the release of chemicals, light pollution, and exploitation pressures. Stock assessment methods could be calibrated and gear biases and estimation of catchability could be quantified in situ using telemetry. Indeed, whole lake telemetry constitutes an excellent opportunity to estimate the otherwise “unmeasurable” (Fig. 2), such as size-dependent mortality, predator-prey interactions (e.g. after stocking of piscivores), or ecosystem reactions to changes in fish populations (e.g. invasions). In this context, the success of common restoration measures, such as biomanipulation [188], depends on risk-sensitive foraging [3], which in the past was indirectly inferred from the capture of fish in gill nets and other gears or was simply inferred from prey responses to introduced predators. Telemetry could be used to directly measure how zooplanktivorous fish respond to stocking of predators, to the removal of fish, to fish-eating birds or otters, or to technological measures (e.g. aeration), or alternatively, how the movements of fish affect turbidity and water quality. Telemetry may also inform eradication of pest species, should this be desired [14].

### Are there simple rules underlying seemingly complex movement patterns and, hence, common drivers for movement across species?

Common rules underlying seemingly complex movement patterns have been identified in a number of aquatic animals, including seabirds, sharks, turtles [117, 269], and freshwater fish [177]. It has been extensively studied how, where, and when individuals move, from which the following common drivers for movement have been suggested: optimal foraging, site fidelity and revisitation, and temporal patterning. For the first, optimal foraging, Lévy walks [269, 299], Brownian motions [129], or similarly simple random walk-type models have been proposed as a simple evolutionary trait that has been adopted by many species when searching for sparsely distributed prey. In recent years, however, this randomness paradigm [200] was the subject of controversial discussions (cf. [28, 234]). In fact, conclusive evidence for the Lévy walk and related hypotheses is still lacking, and it is now regarded as overly simplistic. This perspective has catalyzed a shift towards explaining specific movement paths rather than movement behavior in general [222]. For site fidelity and revisitation patterns, home range or homing affinities have been identified in various freshwater fishes, for which larger individuals were found to generally have larger home ranges [177, 315]. Yet, simple random walk-type models such as (truncated) Lévy walks or Brownian motions are generally inadequate to resolve the patterns [126]. In addition to an individual’s size, the shape of the water body was suggested to affect movement [315], emphasizing how environmental conditions can be regarded as a common driver for movement. For temporal patterns, diel variation as well as daily and seasonal movement patterns, particularly regarding the times of feeding, breeding, aggregating, and resting behavior, have been found in numerous aquatic species [118]. The majority of freshwater fish tend to be predominantly diurnal [17, 52, 58], although marine top predators tend to be more nocturnal [121]. Time is linked to both temperature and photoperiod, which influence the individual’s physiology and motivation for movement. Temperature, for example, has been shown to control activity timing in juvenile salmon [87]. Time and photoperiod can be regarded as a common driver for movement, either affecting movement directly or indirectly by affecting the prey’s behavior, which is then adopted by its predator.

Movement is often assumed to be the result of a single paradigm that neglects its complex nature. An alternative, more comprehensive perspective on movement addresses the animal’s internal state (“why does an animal move?”), its motion (“how does it move?”), and navigation (“when and where does it move?”) capacities, and external factors all interact to generate movement [200]. Lakes provide a nearly ideal environment to collect detailed data that inform complex statistical models and more comprehensive pictures of an animal’s behavior. To fully exploit the complex detection data, powerful statistical methods are needed. Popular models for inferring behavioral patterns from high-resolution bio-logging data include discrete-time hidden Markov models (HMMs; [159, 181, 222]), general state-space models (SSMs; [11, 141, 221]), and diffusion processes (e.g. Ornstein-Uhlenbeck position models or stochastic differential equations; cf. [222] for an overview of the available methods). Fueled by increasingly large and complex telemetry data sets, several methodological extensions towards a more unified picture of movement (cf. [200]) have recently been proposed. For example, hierarchical HMMs provide a versatile framework for jointly inferring movement patterns at multiple time scales (e.g. fine-scale variation in activity vs. coarse-scale migration patterns; [1, 166]), energy budgets and recharge dynamics have been explicitly incorporated into individual-level movement models [125], and group dynamics have been modeled by relating individuals’ movement decisions to herd-level movement patterns [160, 205].

Testing comprehensive models of animal movement in which movement is assumed to be generated by many different factors interacting with each other, against simple null models such as (truncated) Lévy walks, Brownian motions, or related random walk-type models, may provide a promising avenue for confirming (or rejecting) simple rules that have been suggested in the past. This approach can also test the validity of patterns and rules discovered with state-of-art laboratory tracking techniques of aquatic invertebrates (e.g. [59]) for fishes in the wild. In addition, the unprecedented opportunities offered by high-resolution, three-dimensional lake fish telemetry - most notably the possibility to observe an individual’s movement throughout an entire ecosystem at fine temporal resolution while being able to control for multiple variables (Fig. 2) that can affect its behavior in replicated designs, may help to identify new common drivers for movement across species.

### How do learning and memory versus innate behaviours influence movement patterns, including ontogenetic changes?

Animals moving in their natural environments are typically exposed to a variety of factors and conditions that span from highly beneficial (e.g. food or mates) to highly detrimental (e.g. toxic items or predators). The ability of animals to optimize fitness gain by adjusting their movement in response to complexities depends on both innate and learned skills that enable animals to perceive, respond, learn, and remember the structure and dynamics of such factors in their environment. Studies of animal cognition have yielded numerous insights into the mechanisms affecting spatial learning and memory in various taxa [227, 270] and fish in particular [30, 40, 78, 142, 146, 158, 215, 298, 303]. These insights divulged the role of ontogenetic and cognitive processes in shaping movement patterns and their fitness consequences, stressing the critical role of learning from experience during early life. Across species, details were revealed mostly from controlled laboratory experiments on captive animals [30, 215, 270], whereas field studies have been much less frequent, and studies based on movement data collected from free-ranging animals in the wild have been scarce and focused on terrestrial systems (e.g. [108, 218, 219, 267, 285]).

Studies of fish in their natural environment have yielded important insights in the ontogeny of spatial learning and memory. Whereas much of the literature has come from marine species, there is great opportunity to use lakes as a study system to test and advance movement ecology paradigms. Such studies have shown, for example, that the remarkable homing ability of adult salmon depends on long-term olfactory memory of their natal streams learned during early stages of life [113, 259]. Although the basic formulation of this salmon olfactory imprinting hypothesis received further support from later studies and has been broadly accepted, some important details remain controversial [235]. For example, does olfactory imprinting occur exclusively in a limited time (the smolt stage) or at specific sites [259], or as a learned sequence of odors acquired during different early-life stages at different times and sites [114]? Furthermore, fish might learn other cues and in a more complex manner. For example, juvenile reef fish responded to cues sensed through different mechanisms (olfaction, hearing and vision) at different sites experienced during their early-life movements [128]. Tracking fish movements throughout their life cycle, and especially during early stages of life, offers a unique opportunity to tackle such complexities. Earlier studies of fish movement mechanisms have used boats to follow individual fish marked by a tethered float [111], ultrasonic [112], or radio [14] tags, resulting in relatively limited datasets of few individuals tracked at low frequency and for short durations. Although these studies made some important propositions – that wild white bass (*Morone chrysops*) can swim directly homeward in open water presumably by using a sun compass [111] and other cues [112], and that wild carps can quickly learn and remember the location of new food resources [14] – more conclusive insights and more in-depth investigation of the mechanisms underlying the observed tracks were still rather limited. This powerful research system has just started to be applied to study topics related to ontogeny of spatial learning and memory. Topics strongly related to ontogeny, movement, and spatial learning and memory, such as personality traits [2], cognitive flexibility, and inter-individual variation in space use [174], time-place associations [241], landmark use [303], and various other orientation and navigation mechanisms [31], have been predominantly studied in the laboratory, and now can be critically advanced by implementing high-throughput field telemetry approaches.

Understanding how early-life processes shape animal movement and behavior through learning and memory is also important for managing populations, for example of fishes in lakes and rivers. Better understanding of these processes can guide the development of infrastructure to facilitate fish migration and survival in light of anthropogenic disturbances such as river dams [100], or by enriching the relevant early-life environment of captive-reared fish [142]. Studies examining fish response to capture by hooks can also largely benefit from high-resolution fish tracking. For example, movements of both fish and fisher might be tracked rather exhaustively in a closed lake system, to accurately estimate the probability of captures and encounters and to elucidate the factors affecting these probabilities [7, 164, 193]. More generally, high-throughput wildlife tracking systems such as acoustic telemetry in lakes can unravel some of the most basic relationships between animal cognition/memory and movement (Fig. 2). This has recently been shown through the use of ATLAS, a new reverse-GPS tracking system that is principally very similar to acoustic lake telemetry, to reveal the first field evidence for a cognitive map and spatial memory of multiple specific targets by free-ranging animals within their large (100 km^2^) natural foraging area (Toledo et al. [285]). Furthermore, such tracking projects can be coupled with methods providing complementary information on behavioral, physiological and environmental changes, as well as experimental manipulations of learning and memory by altering landmarks, fishing habits (e.g. bait type), sensory cues, and the presence of informed vs. naïve fish.

### To what degree do social interactions influence movements?

**Fig. 3:**
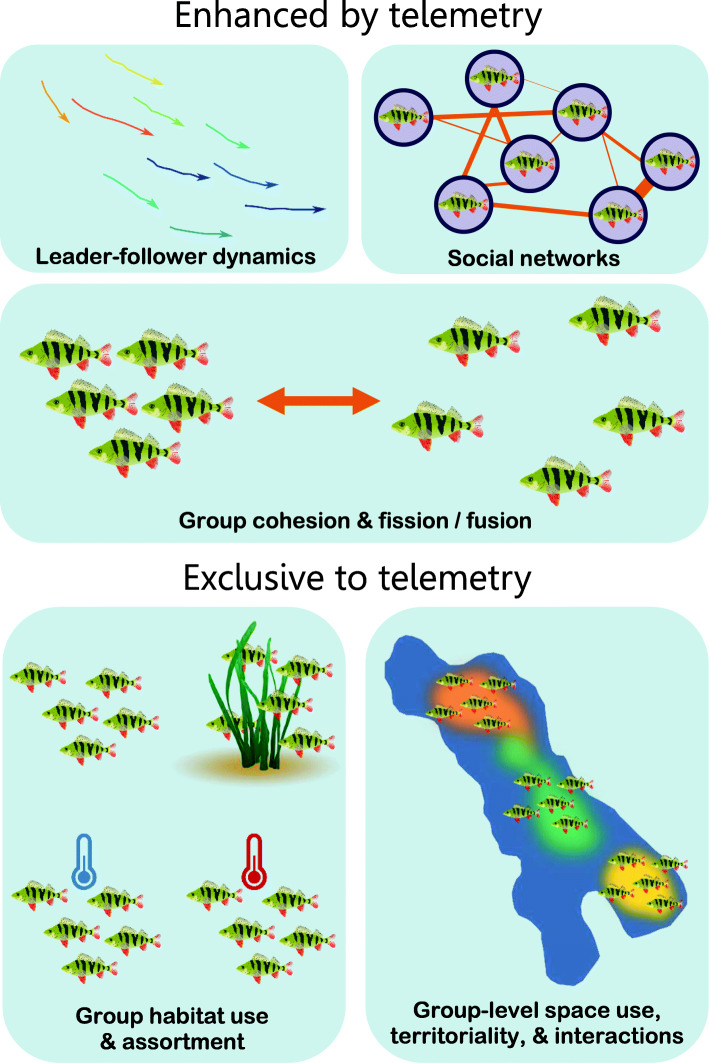
Data from acoustic telemetry will greatly enhance our analysis of social and collective behaviour in fish, as well as allow new forms of analysis that have previously been impossible in the wild. The analysis of leader-follower dynamics, social networks, and group cohesion can now be performed at much greater temporal and spatial scales using telemetry data. This will allow study of how these social factors affect ecological phenomena including group foraging, migrations, and predator avoidance, and how changing environments further modulate these effects. Telemetry data will revolutionize the study of the interactions between habitat use (e.g. in response to physical structure or factors such as temperature of oxygen availability) and passive and active assortment of phenotypes among and within groups. In addition, an opportunity now exists to examine among-group variation in space use, territoriality, and changes in social group membership, with possible effects on individual fitness

The study of animal social behaviour is fundamental to our understanding of behavioural, physiological, and evolutionary ecology. Group living is key for predator-avoidance, foraging, and reproduction in most animal taxa. This directly affects organismal fitness but also modulates the outcome of numerous life-history and evolutionary trade-offs. There is also increasing evidence that sociality plays a key role in the maintenance or erosion of within-species phenotypic variation in behavioural and physiological traits [140]. Fish display numerous forms of complex social behaviours including social networks, dominance hierarchies, social learning, and coordinated group movements with leader-follower dynamics (Fig. 3) As such, fish are often used as models to study animal social behaviour and form the basis of a large proportion of our knowledge about emergent group behaviours. Notably, however, most of this research with fish has been done in the laboratory, mainly because of the extreme difficulty associated with long-term measurements of individual fish behaviour in the wild [147]. Our knowledge of how fish social groups function in the wild, and how they are affected by environmental conditions, has therefore been hindered by this basic constraint in our research capabilities [103].

Tools are now available to begin addressing detailed questions of social interactions and animal movement. Lab-based observations of fish social behaviour can be realized with sophisticated software for automatically tracking the trajectories of multiple individuals from recorded video [226, 245]. These data are a series of x-y coordinates for each individual within a group that can in turn be used to quantify: 1) group-level metrics, including group cohesion and polarity; 2) the behaviour of individuals within groups such as individual speed, alignment, spatial positioning, distance from group mates, and social network position; and 3) the propagation of changes in movement metrics throughout social groups. The spatial and temporal resolution of telemetry systems in the field can now advance basic forms of these analyses on freely roaming fish in their natural habitats, with the coordinates of individual detections being analogous to the x-y coordinates captured by automated software in laboratory behavioural arenas. Analysis of movement propagation in the lab is used to inform leader-follower dynamics in fish social groups ([144]; Fig. 3), and in the wild could provide information on migrations and other phenomena related to collective movement [25, 306]. Telemetry data are currently being used to infer differences in individual space use and habitat preferences within species [83, 199], but it is highly likely that these are also affected by social dynamics in ways that we are yet to understand but that will now be possible. Increased knowledge of fish social systems will also provide knowledge on how group movement and behaviour affect individual vulnerability to different fishing methods [122, 287]. Perhaps most importantly, increased knowledge of fish social systems in the wild will help us understand their responses to natural and human-associated changes in environmental factors such as temperature, oxygen availability, turbidity, and food availability. A promising opportunity also exists to combine telemetry movement data with other forms of logged or transmitted data from individual fish (e.g. heart rate data, temperature) to carefully dissect the interplay among animal movements, their social environment, their physiological state, and the external environment [65, 66]. It will also be possible to combine all of this information with established theoretical movement models from lab-based work to more fully understand fish social dynamics, emergent group behaviours, and then predict their responses in the wild and empirically test these predictions.

Despite these exciting opportunities, there remain many challenges that must be addressed before we can fully take advantage of acoustic telemetry in the study of fish social behaviour. In order to fully understand how fish are being influenced by their social environment, we must have data for all or at least the vast majority of fish within a natural system. This is extremely difficult because in most cases it will be impossible to know if all fish within a system have been captured and tagged. A possible solution may be the removal of most fish, followed by stocking with a known number of tagged individuals, or the use of dedicated fishless lakes or artificial ponds. An additional challenge will be the development of a statistical and analytical framework for studying the desired social behaviours and emergent phenomena. To be most useful for social analyses, telemetry data must have a high spatial and temporal resolution and low error. Lab-based work can provide precise positions of individual fish dozens of times per second [226, 245]. This is not possible with even the most advanced forms of acoustic telemetry, and so we will need to work back to uncover the minimal adequate spatial and temporal resolutions needed for basic analyses of individual interactions, spatial positioning within groups, and group fission-fusion processes. Enhanced resolution also greatly increases the required computing power and analyses time, and so it might initially only be possible to perform the most sophisticated analyses on subsets of data.

### How does the distribution of prey impact movement?

Prey distribution and availability can highly alter the behaviour and movement of predators [19]. Initially, predation concepts focused on the optimality of foraging behaviour, i.e. maximization of the rate of energy intake, in relation to prey density and distribution [175]. Later, predation risk [305], competition among conspecifics [88], effects of environmental abiotic factors [4], level of individual [290], or individual state [185] were introduced into the models explaining the effects of prey on predator distribution. These concepts mostly targeted ultimate causes of predator-prey distribution interactions and their effect on life history traits and fitness of both predators and prey [96]. Concepts such as optimal foraging, game theory and ideal free distribution further considered that individuals tend to optimize their foraging strategies based on all relevant environmental factors (such as the amount of prey, predation risk, and number of conspecifics) and internal physiological state, and chose the behaviour that maximizes individual fitness and future reproduction [96]. However, it is now widely recognized that wild animals are limited by incomplete information and imperfect ability to analyze information and foresee consequences of alternative behavioural options [9]. Consequently, recent research has shifted more towards individual level and proximate causes of predator-prey distribution interactions.

Much attention has been given to the role of different decision-making processes for involving individual decisions and their regulation into foraging behavior [9, 200]. Current thinking frames individuals as units determined by various properties (individual genotype, physiological state, age, or size) and moving in space defined by multilevel landscapes of, e.g., fear [162] or energy [246]. Individual space use then depends on the overlay of these landscape ‘bricks’ (e.g., infrequent use of locations with rich food and high predation risk) and actual cognitive and physiological state of an individual [89, 101]. Both individual state and landscape topography are affected by environmental factors (e.g. temperature and light in aquatic environments) and change dynamically in time [89]. Yet, many important questions are poorly understood in these fields and high-resolution movement data can be a key component in their understanding: e.g. proper matching of the landscapes of fear and energy with resulting movement trajectory [89]; effects of prey availability on predator behaviour under different environmental contexts [47]; predator-prey personality interactions in forage/escape behaviour; mismatch in the timing of predator-prey activity peaks [8]; temporal individual variation in the forage/hide behaviour, the role of individual traits in ontogenetic shifts in space and resource use [197, 242]; and the causes and triggers of diel vertical and horizontal migrations [186, 242, 250].

Even relatively large water bodies up to several hundreds of hectares can be fully covered by positioning systems [20, 307] to provide fine-scale positioning of both predator and prey over long periods of time that can be used to answer a variety of questions related to predator-prey interactions. For example, Jacobsen et al. [134] identified alternative foraging strategies in acoustically tagged Eurasian perch in mesotrophic and hypereutrophic conditions. In a long-term movement study, Nakayama et al. [198] found distinct diel horizontal migration of Eurasian perch likely related to foraging opportunities. Baktoft et al. [16] used tagged Eurasian perch to quantify the links between metabolic rate and activity patterns. Kobler et al. [150] studied behavioural types of pike using radio-telemetry in a lake and found distinct differences in habitat use and activity levels, which they related to an ideal free distribution pattern. Madenjian et al. [176] demonstrated a positive effect of food availability on consumption rate in walleye *Sander vitreus*. In the same species, Raby et al. [237] concluded that drivers such as temperature and food availability influence migratory behaviour. All these studies show the high potential of telemetry in studying predator and prey space use and their spatial interactions. We believe that the development of high-resolution telemetry and statistical techniques to identify and analyze patterns in multidimensional big data will help understand predator-prey interactions in great detail. Current technology of high-resolution tracking dramatically expands our abilities to uncover predator-prey spatiotemporal overlay and use it to infer their direct and indirect interactions (Fig. 2). Such sampling can be accompanied with measurements of suitable individual traits before or after tracking and use these traits in possible proximate or ultimate explanations of their behavioural strategies and predator-prey interaction strengths. The main limitation for such studies currently seems to be the need for a carefully planned protocol with a large number of tracked fish to obtain robust patterns.

### What sensory information do animals use to sense prey, breeding partners, and environmental conditions?

The sensory perception of the abiotic and biotic environment is the basic input for fish behaviour. Fish may use a wide array of senses (gustation, olfaction, vision, lateral line, hearing, magnetoreception, and electroreception) for orientation in the environment and one or multiple senses may be used as a basis for their behavioural decisions. To disentangle which sensory system is used for assessing particular situations, experimental designs using sensory blocking, nerve suppression, nerve transection or ablation experiments are frequently used [201, 213, 229]. Sensory ecology of aquatic organisms is predominantly studied under controlled laboratory conditions [196, 279]. Due to the ability to precisely track animal movement beyond laboratory environments, novel research designs using 3D telemetry technologies have the potential to shed light on many research topics dealing with sensory perception important to predator-prey interactions, communication among conspecifics, and animal orientation within the visually limited space of aquatic environments. Such a design was implemented to discriminate among visual, magnetic and olfactory navigation to natal stream in sockeye and masu salmons *Oncorhynchus nerka* and *O. masou* [291].

The ability to precisely track individual fish opens new opportunities to test hypotheses and validate laboratory findings linking sensory information to individual behaviour (e.g., [59]) in ponds and lakes. Two approaches can be used for experimental study designs in lakes: manipulation with the environment and manipulation with fish physiology and sensory ability. Using multiple small lakes or ponds (or dividing them with curtains) and manipulating variables (e.g. turbidity, anthropogenic noise, pH or light pollution) may help disentangle the effect of tested variables on the fish behaviour and fitness [263]. Study design may alternatively involve manipulation with fish physiology by using slow-release implants and comparing it to non-manipulated individuals [180, 182]. Finally, experimental designs using sensory blocking, nerve suppression, nerve transection or ablation experiments may help determine which sense provides critical input for the observed behaviour. Novel approaches using depth, temperature, acceleration, predation, or metabolism-level sensors may be integrated in the study design, thereby enabling a wider interpretation of the data [5, 106, 238].

Study designs using 3D telemetry to differentiate among senses used for observed behaviour would require careful study design using one of the above-mentioned options. As an example of such an experiment in semi-wild conditions, disabling a selected sensory input in selected prey individuals and comparing them to controls may help disentangle the role of sensory information in predator avoidance and quantify the role of each sensory input. Manipulation of the sensory ability of predators can be used to discriminate which senses are important in which part of the predator-prey cycle [201, 229]. Uncertainty in the data interpretation may be further minimized by monitoring all potential prey and predator individuals. Given the cost limitations, preference should be given to simple systems with limited predator-prey species interactions to enable thorough interpretation of the results and to minimize the risk of study failure [170]. While we argue above that purely behavioural studies would benefit from as many tagged fish as possible, we partly take the opposite stance here because experiments targeting sensory information are potentially of an invasive nature. Such experiments should be planned carefully to minimize the number of individuals used for the study and maximize their welfare [38, 247]. Therefore, the questions should be addressed primarily using non-invasive methods such as environmental manipulation or temporary sensory suppression by chemical treatment [201]. Joint efforts of physiologists and behavioral ecologists respecting these limitations can still provide novel insights in the use of sensory information in fish behaviour in lakes.

### Can movement data provide information on the ecosystem role of megafauna?

Ecosystems are built upon matter and energy, the movement of which generates ecosystem services [69]. In lakes, matter and energy cycle among riparian, benthic, littoral, and pelagic zones; gravity and flow create connections but organismal movement is critical to creating linkages and generating ecosystem services. Rates at which these processes occur vary as a function of a variety of factors operating at broad spatial scales such as those driven by temperature as well as shorter scales such as depth and nutrient loads [264]. Organisms carry out ecosystem services by cycling matter and energy through their bodies, as such, they develop functional roles in the ecosystem as producers, consumers, decomposers, etc. [22, 123]. Valuable research has been carried out in lakes to reveal relationships among lake morphology, productivity, and fish biomass (e.g. [51, 265]) and with telemetry tools we have the capacity to expand this knowledge with finer-scale details of the functional roles that fish have in these systems and the feedbacks between consumers and producers in the ecosystem. Throughout the field of ecology, there is broad interest in understanding how roles are partitioned among species in an ecosystem, and how the system responds under stress such as when challenged by invasive species, climate change, or pollution. Understanding roles and identifying pathways through which ecosystem services are generated is therefore a key question to ecology, albeit one that has been afforded less consideration in the context of movement ecology [117]. In lakes, productivity scales with the perimeter/area ratio, suggesting that small lakes, rather than great lakes or seas, would be ideal venues for investigating habitat coupling and ecological roles with replicated whole lake experiments including manipulations of the fish assemblage and experimental alterations of lake productivity [257, 307].

Whole-lake studies have contributed in substantial ways to our understanding of energy landscapes and ecosystem services. Predation and competition are the key biotic processes that structure lake fish communities and manipulative experiments in lakes have illuminated how these processes operate [133]. Replicated whole-lake experiments have been conducted by modifying the fish community and observing changes in abundance and growth to reveal mechanisms that structure assemblages (e.g. [46, 51]). However, existing studies have lacked the resolution to observe competition and predation in situ. Manipulative experiments in whole lakes provide ideal templates for research on ecosystem roles when coupled with tools that allow direct inference of material and energy cycling, such as stable isotopes [294] or chlorophyll measurements in situ [51]. Stable isotopes have revealed transmission of carbon and nitrogen within lakes and the terrestrial-aquatic interface [220] as well as shifts in the trophic network as a consequence of species invasions [293]. Measurement of stable isotopes linked with movement data can illustrate how matter is transferred within the lake and what functional movement classes exist within species and whether movement syndromes (i.e. consistent individual differences) exist. Movement syndromes may be key to determining how intraspecific differences in behaviour drive ecosystem roles. Acoustic telemetry in replicated whole-lake experiments will reveal how individuals, populations, and communities shift their patterns of space use across days, seasons, and years to incorporate and deposit matter and energy within their confined landscape. Layering this information with abiotic data will reveal drivers of migration and dispersal within habitats across time scales [22, 37]. We can then link where and when animals move with the consequences of that movement for the ecosystem, established from site-specific sampling of lake productivity and contrasts among species under investigation. Multispecies studies in whole lakes can also reveal dynamic niche partitioning and species interactions including predation, competition, and parasitism when multiple species are tagged (Fig. 2). Critical to this is considering scale by contrasting results from lakes of different size: we will likely find increased sympatry and decreased connectivity with increasing habitat size, a factor that can easily be investigated in these closed systems [133].

We envision replicated whole-lake experiments that specifically investigate multi-species dynamics in habitat use and the nature of connectivity within lake ecosystems. Instrumented individuals moving within an array of acoustic receivers will reveal patterns and drivers of movement across spatial, temporal, and ontogenic scales. Spatial overlap of individuals and species can be calculated using kernel density or convex hulls from two- or three-dimensional positions within arrays (e.g. [104]; Fig. 2). Detection data from acoustic receivers can be investigated using network analysis (Fig. 3) to determine which species are central to connecting the ecosystem across space and time [136] and functional movement classes can be identified within and across systems from cluster analysis [35]. Contextual data can be derived from biologging sensors including accelerometers that measure fine-scale behaviours that can be interpreted as foraging or reproduction to reveal the frequency and spatiotemporal distribution of these exchanges of matter and energy (e.g. [43, 289]). Novel tag sensors and analytical models can also be used to remotely reveal predation in lakes with smaller risk of a predator evading detection than in marine systems but the tag size still limits the size of fish that can be studied [93, 106]. Telemetry data can predominantly be derived from fish but interactions with other species such as ducks [209], crayfishes [308], semi-aquatic mustelids, turtles, frogs, snakes, or crocodilians are also certain to be important and some of these species could be tagged as part of a broad community study. Investigating movement responses of fish to experimental manipulations such as nutrient subsidy (e.g. [220]), introduction of novel species [46], change in water quality (e.g. temperature, clarity, pH) can then be used to establish mechanisms explaining movements observed in telemetry data. Replicated experimental designs will be critical to establish causality and determine whether movement phenotypes drive ecosystem services or whether characteristics of the ecosystem shape the movements of animals that reside within.

### How much does the physical environment influence movement?

Ecologists are continually searching for fundamental patterns of movement that are predictable across organisms and scales [269]. One encompassing pattern deals with how much an environment influences movement patterns, and whether collected trajectories are representative of an animal’s full potential for movement [12, 21, 35]. Movement data for such comparative problems are typically collected from a wide range of environments that are often assumed comparable rather than explicitly tested. These limitations are an artefact of early movement tracking technologies and their relatively small sample sizes, whereas contemporary technology allows for greater scalability and replication. Many of the largest lakes on the planet have hosted extensive tracking networks, suggesting that the gap between technology and scale-appropriate studies continues to narrow. But there is ample room to investigate ecological phenomena at smaller scales that encompass a greater diversity of lake types and ages and thus physical environments [133]. Such a broad variety of smaller and usually self-contained ecosystems gives researchers the ability to perform either observational or experimental studies. The field of limnology consistently takes full advantage of small lake attributes to investigate fundamental patterns of abiotic interactions (e.g., biological, chemical, and physical). The morphometry of smaller lakes can range from simple gradual depressions with circular boundaries to complex depth profiles with asymmetrical boundaries. Where a lake is located will affect how its morphometry limits utilization of light and thus thermal input and stratification. There are many other physical environment modifiers (e.g., wind, geothermal, underwater springs) that can also be influenced by location and have the potential to affect fish movement. Uncovering how the physical environment influences organismal movement across and within gradients of change (e.g., aging, disturbances) is another avenue to consider that is also understudied. In summary, lakes can provide the necessary scalability to investigate the relations between physical environment and movement, through both observational and experimental means in stable or dynamic contexts.

There are relatively few lake studies that specifically examine the physical environment using telemetry and even fewer that study multiple lakes simultaneously. Often, studies will characterize an entire lake’s physical environment (e.g., temperature, light) with relatively coarse sampling resolution, either spatially or temporally. Yet, lakes are perfect arenas for detailed fine-scale sampling of processes that cannot easily be detected in the vast marine environment. Gerking [91] described the variability of individual fish movement behaviour as an association between an individual and its surroundings that is informed by sensory stimuli and driven by recognition of familiar areas. A more modern perspective also suggests that physical environments often contain recognizable landmarks so fish can learn and generate spatial maps [31]. What is not clear is what drives shifts in fish home ranges, which stimuli inform movements more than others, and how to respond to changes – all as a function of their physical environment. At a coarse scale, studies have shown that fish can consistently find the same food patches, discriminate among habitats using multiple cues, and optimize foraging strategies in heterogeneous physical environments [30, 128, 211]. Interestingly, when multiple connected lakes are considered, fish dispersal seems to be more affected by spatial distribution of lakes, number of connections, and suitability of corridors as opposed to local environmental factors [27, 216]. At a finer scale, studies have shown that lake morphology (simple basin vs. complex) can influence habitat use, spatial distribution, and activity [239]. Furthermore, lakes with stratification can influence vertical movement patterns [102, 208]. As understanding of individual lakes and their physical characteristics continues to grow, so too will the opportunities to link such phenomena with fish movement ecology.

Lakes are ideal for revealing relationships between the physical environment and animal movement, particularly when considering using multiple lakes simultaneously. There are unlimited ways to design movement studies using lakes but to disentangle the physical environment from organismal movement, we have four recommendations. Our recommendations consist of different types of studies 1) before and after, 2) gradients (longitudinal or latitudinal), 3) replicated, and 4) stable vs. dynamic comparison. Before and after type studies can take a lake or multiple lakes monitored before and after some ecological phenomenon, alteration in lake morphology, or physical change occurs but the sample unit is the lake (e.g., some lakes are controlled while others represent treatments). The second is the same but the sample unit is the lake in a nested design (e.g., the lake is subdivided with an impermeable barrier). Often, these studies emphasize using lakes with similar physical characteristics and are in close proximity of one another. Longitudinal and latitudinal gradients are simply studies where lake choice is spread along a coordinate axis (e.g., north-south, east-west) so variations of light and thermal regimes can be incorporated. These studies are characterized by long distances between study areas where each lake is arranged at the furthest and opposite edges of the study organism’s distribution. For example, one lake in this study may be affected by ice coverage in winter while another lake in the study has year-round open water. Additionally, gradients along elevations are also possible. The third recommendation is focused on lakes where anthropogenic activity manipulates the physical environment intermittently or frequently to introduce altered physical environments. Examples of alterations include but are not limited to different forms of pollution (e.g light, sound), boating traffic and shipping, habitat modification (e.g., aeration, weed removal, shoreline development, thermal effluent). Comparing the differences between altered and unaltered environments is particularly suitable for urban areas. Alternative disturbances could be drought and severe water level decrease, prolonged ice coverage and increased ice thickness, hypoxic events driven by algal blooms, and introduction of an invasive species that specifically modifies the physical environment. Overall, all the recommendations here only scratch the surface of possibilities but provide a template for an unexplored research area that can be enhanced with other experimental design techniques such as transplanting fish and manipulating physical environments.

### How will climate change impact animal movements?

Climate change is a ubiquitous process affecting all ecosystems and one of the major drivers of species extinctions [132, 292]. In response to climatic change, geographic range and distribution shifts have been observed in a number of species [161, 277]. Ectotherms are particularly sensitive to environmental temperature extremes [231], explaining the conformity found between their latitudinal ranges and thermal tolerance [277]. In freshwater teleosts, moving away and dispersing to find a more suitable environment, matching with their own biological constraints, is indeed commonly observed in response to climatic change [62], with a general tendency of range contractions at warm range edges and shifts to higher altitudes or latitudes [277]. However, animals are constrained by system boundaries with limited opportunities to disperse and relying upon alternative strategies to cope with climate change [61]. This is especially true for lake teleosts, for which climate-induced changes of lake properties and phenology, such as catchment hydrology, lake ice phenology, thermal characteristics, nutrient supply and cycling, primary production, and bacterial blooms [92] can create challenging conditions for development and survival. Additionally, climatic effects often coincide with other anthropogenic stressors affecting lake ecosystems such as eutrophication, pollution, biological invasions, habitat degradation, and direct exploitation of organisms [51, 109].

Beyond distribution shifts, teleosts strongly rely on their phenotypic plasticity, i.e. ability to adjust their behavior and physiology, to cope with new climatic regimes and associated ecosystem changes (for a general review see [24]), in particular, under a rapid climate change that is limiting the capacity for evolutionary adaptation [295]. Changes in the abiotic environment can directly affect the metabolic processes of fish, more specifically, warming water temperatures accelerates metabolic rates leading to an overall increase in the demand for energy. Fishes can acclimate to warming conditions by metabolic thermal compensation of resting cardiorespiratory functions [252]. Metabolic plasticity and thermal compensation to extreme temperatures carries implications on performance and fitness-related traits, such as cognition [318] or predation rates [266], although the extent to which thermal plasticity affects animals in the wild is largely unknown. In addition to physiological adjustments, fish can modify their habitat use and activity patterns to avoid additional energetic costs of sub-optimal environments. To escape from warming waters in summer, fish exhibit behavioural thermoregulation [271]. Alternatively, fish may also reduce energetically costly behaviours and overall activity when environmental temperatures exceed their thermal optimum. Water temperature also drives spawning migrations, with inter-annual variations of water temperature affecting their timing [154, 282] but also the propensity of migrations among partial migrants [34, 36]. Finally, climate change is not restricted to changes in temperature, and alterations of other physico-chemical water properties can be expected to challenge the survival and persistence of lake teleost fish, such as levels of dissolved oxygen, pH, and load of dissolved and particulate organic matter [165]. Further, changes in general energy fluxes, habitat use and phenology in fish will also impact interactions within and among species, such as prey-predator interactions and the spread of invasive species, possibly inducing feedback effects on ecosystem functioning by affecting lower trophic levels and nutrient cycling (see section on the Ecosystem Role of Marine Megafauna) and ultimately causing regime shifts [36]. Research shows for instance that changes in hydrology and temperature will favour the spread of warm-affinity and temperature tolerant species [13, 245].

Under the strong influence of terrestrial and atmospheric inputs, freshwater ecosystems are sensitive to climatic changes [79] and considered the sentinels of global climate change [314]. Small lake systems could therefore serve as sentinels of the risk associated with climate change and provide unique information on the capacities of ectotherms to adjust and adapt to changing environments. Plastic changes of labile phenotypic traits being the first line of action to changing environmental conditions [24], fine-scale animal tracking in small whole-lake systems can provide a window into plastic responses of animals and their capacity for adaptation, but also an alert system predicting the effects of climate change on natural populations and ecosystems. We therefore suggest multi-species tracking in replicated whole-lake systems along a latitudinal gradient covering the geographic range of sentinel teleost species. The study of animal movements in whole-lake systems in combination with the bio-logging of key physiological functions (e.g. accelerometer and heart-rate loggers – see section on Physiology) will inform us on the behavioural and physiological adjustments to changing environments before the effects on lifetime fitness and patterns of natural selection and evolutionary change become evident. Given the difficulty to measure effects of climate change over relatively brief timescales inherent to single-lake telemetry studies, such studies could be replicated and carried out along climate gradients following a Space-for-Time Substitution approach that can provide novel insights on the effects of climate change on wildlife [189]. Such an approach could also be complemented by manipulative studies in relatively small mesocosm or pond systems to test specific climatic scenarios [280], which would further contribute to our understanding of the plastic and adaptive responses of teleosts to climate change.

### How can risks, consequences and benefits of biologging at the level of individuals and populations be evaluated?

Fish are widely used animal models in research and are a frequent subject of telemetry research; consequently, increasing attention is paid to the tagging protocols [33, 65, 66, 97, 316]. Evaluations and testing address all stages from capture to release [284] and even post-tagging evaluation [41, 97, 300]. Evidence of nociceptory perception by fish suggests that caution must be devoted to these tagging procedures [38] that can impair their welfare and introduce bias in experiments when data reflect distressed, injured, or otherwise inauthentic movements. It is therefore of utmost importance to ensure that best surgical practices are being used [40, 248]. As such, numerous studies on the effects of analgesics, substances dedicated to reduce or suppress the pain sensation, have been conducted on fish (e.g. [73, 191, 248, 316]) and recently reviewed by Chatigny et al. [73]. Previously, the impact of the tag size and weight relative to fish weight had also been debated [41, 137, 272, 311]. To determine the limits and avoid negative effects on the behaviour, the 2% limit (tag weight over fish weight in the air) from Winter [311] was adopted as a general rule for a long time. The handling of fish and implantation of transmitters on or into fish inevitably raises the question of tagging effects [53]. It is especially challenging to study this for aquatic animals in the wild because visual observations are often impractical and complex and it is almost impossible to gather data on fish behaviour without handling and/or biologging. Most studies have been conducted in laboratories but these can underestimate tagging effects as fish do not have to cope with natural stressors [302]. Jepsen et al. [137] reviewed the effects of external electronic tags on fish and listed the following that have been addressed in the literature: retention/expulsion, survival, infections/wounds/tissue reactions/healing, general behaviour/activity, swimming performance, feeding, growth, migration, equilibrium, physiological effects, buoyancy, predation, catchability, social interactions, reproduction, and responses to transmitter output.

There are a growing number of laboratory-based studies that evaluate various aspects of the surgical implantation of electronic tags (reviewed in [54]). For example, Brown et al.[41] tested the impact of tag weight on swimming performance of juvenile rainbow trout (*Oncorhynchus mykiss*) implanted with radio transmitters in a Blazka type chamber [273]: the swimming performance was not altered up to tags representing 6–12% of the body weight. Wagner and Stevens [301] tested the effect of intra-peritoneal transmitters and sutures on swimming behaviour (number of C-turns and sprints, total distance travelled) of rainbow trout 3 weeks after surgery. Control fish were also anaesthetized and handled but did not undergo surgery, which apparently had no effects on behaviour. Newby et al. [202] showed in chambers that PIT tagging had no short-term effects on the feeding behavior of juvenile rainbow trout. They also tested their swimming performance (time to fatigue) by comparing a pool of individuals tagged 40 days before the experiment, for which the wound had healed, with another group tagged on the day of the experiment; they found no significant differences. Harms et al. [110] tested the effects of analgesics on koi (*Cyprinus rubrofuscus*), both on behaviour in tanks and on clinical changes. Except for one of the applied analgesics, all fish that had surgery showed reduced activity, deeper position in the water column, and decreased feeding activity; they also exhibited clinical pathology changes. In the field, Wilson et al. [313] compared downstream spawning movement of walleye tagged in a given season to individuals tagged in previous years in lakes. Fish tagged in a given season travelled slower downstream from the river spawning sites. Jepsen [138] provided evidence that growth and survival of radio-tagged pikeperch was not altered over the long-term. Handling is also part of the tagging process. Baktoft et al. [23] concluded that pike handling had only a transitory effect on the activity level and this effect was not detectable 48 h post-release.

In the future, we expect that technological advances in transmitter miniaturization ([171], Nishiumi et al. 2018) and injectability [171] will diminish both risks and impacts associated with tagging. Addressing issues with tag expulsion will also be important to improve resolution of fates. Nevertheless, the greatest care must be devoted to tagging protocols and fish welfare in order to reduce to the largest extent possible the tagging effects and ensure reliable results from experiments. Moreover, attention should be paid to short-term impacts of tagging, by comparing behaviours in the days following the release to later periods when the wound has healed; longer-term effects can also be tackled by comparing fish tagged over longer periods. This is not a perfect solution because it assumes that the control tagged fish are not impacted by tagging, but it may still be a good compromise where untagged fish cannot be tracked reliably. Ultimately, the benefits of telemetry studies must be weighed against the potential costs, emphasizing the importance of fish welfare by mitigating negative effects and refining handling protocols for optimal research validity.

### How do we integrate physiological context into tagging studies to gain a more synoptic picture of movement and behaviour?

Effective species management requires detailed knowledge of focal species’ movements and patterns of habitat use [63, 71]. Biotelemetry and biologging are powerful tools in this regard, permitting the construction of models assessing population and community-level processes from information gleaned from individual animals [65, 68]. However, accurately predicting the response of animal populations to environmental change [161] requires mechanistic links between the environment and population-level processes [143, 225, 261, 283]. Individual-level processes are governed by physiological responses to environmental conditions determining individual fitness and performance [64, 127]. In fish, much of the work deriving physiological links between the environment and population-level processes applies the frameworks of metabolic/aerobic scope (AS), or dynamic energy budgets (DEB [72, 179, 283]). AS and DEB approaches rely on compartmentalising different physiological processes within budgets determined by the capacity of an organism to supply the required oxygen and/or energy, to fuel those processes. In both cases, elevated metabolic costs associated with persisting in energetically demanding [183] or otherwise suboptimal environmental conditions can reduce resources available for other processes, reducing fitness. Environmental modulation of basal metabolic costs could therefore drive patterns of movement and habitat selection in fish as individuals select habitats where metabolic scope and scope for activity are maximised [124], or otherwise avoid engaging in energetically costly behaviours in conditions where metabolic budgets are reduced [269, 309]. Both AS and DEB approaches linking physiology with population level processes often rely on the results of laboratory experiments, and so suffer from the difficulty of transferring these findings to wild animals in natural environments. There is a paucity of information regarding the ecological consequences of intraspecific variation in physiological traits, how changes in the physiological status of fish influence the relative importance of environmental conditions in determining fitness, and how behavioural responses to environmental stress may modulate their impact. While many physiological measurements and biomarkers are currently restricted to quantification under lab conditions, advances in biologging and biotelemetry allow us to better understand the ecological relevance of these biomarkers, and even measure them in free swimming fish [65, 190, 288].

Complete coverage of lakes by high-resolution acoustic telemetry arrays allows continuous monitoring of fish at the fine spatiotemporal scales [16] required to identify rapid or infrequent behaviours that contribute significantly to metabolic demand [43, 143]. Lakes also restrict the dispersal of fish, maximising opportunities for retrieval of biologgers [65] and facilitating repeated measures of physiological traits in recaptured wild fish [206]. Multi-sensor approaches for monitoring the environment, physiological state, and movement of fish provide direct indications of the role of physiological processes in determining patterns of movement and behaviour. Lucas et al. [173] recorded heart rate and fish position alongside environmental temperatures to estimate metabolic rates in free swimming pike and partition the metabolic costs incurred by activity, digestion, and water temperature. The majority of work incorporating physiology into fish movement studies has been conducted in non-lake environments, but used approaches readily applicable to lake systems. Brownscombe et al. [44, 45] calibrated accelerometer-derived measures of activity with metabolic demand in bonefish (*Albula vulpes*) using swim-tunnel respirometry, and subsequently monitored fish energy expenditure across habitat types and environmental gradients, whereas Slavik et al. [274] were able to assess the energetic costs associated with specific patterns of space use in catfish (*Silurus glanis*) using a combination of positional and electro-myocardiogram (EMG) telemetry. Data loggers concurrently recording heart rate, temperature and acceleration (e.g. DST centi-HRT ACT logger, Star-Oddi) implanted in fish alongside an acoustic transmitter would allow simultaneous estimates of fish position, activity, heart rate, and ambient temperature within a telemetry array. Multisensor approaches such as this have been successfully employed to record physiological and behavioural stress responses in response to seismic air gun noise in Atlantic cod (Gadus morhua) [75]. Laboratory calibration of sensor outputs with measures of energetic demand would strengthen these approaches further. Comparing patterns of movement in the wild to lab-derived physiological traits measured in focal fish is an alternative way to incorporate a physiological context in fish telemetry studies. Baktoft et al. [16] implemented a combination of lab-based respirometry and high-resolution acoustic telemetry to investigate links among phenotypic variation in metabolic rate and swimming activity in wild perch. No relationships between traits were found, providing evidence that links among physiology and patterns of movement may not manifest as predictively as some theoretical frameworks proclaim (e.g. Pörtner [230]), and this work remains one of the few attempts to test the relevance of such concepts in the wild.

Whereas metabolic traits are relevant to the study of animal movement, and standardised approaches to their measurement improve comparability among systems [55], there are alternative physiological traits that can be measured. For example, testing the resilience of individual fish to environmental extremes in laboratory conditions [195], and subsequently monitoring their movements and behaviour across environmental gradients in the wild could contextualise the role of physiological performance constraints in shaping habitat use. Relevant physiological biomarkers may also be provided by tissue and blood sampling. For example, enzyme activities (e.g. citrate synthase, lactate dehydrogenase) in metabolically important tissues such as the heart or liver can provide indications of fish’s aerobic and anaerobic metabolic capacities [207], and so may be relevant for determining how fish utilise habitats with differing oxygen availability. Similarly, traits related to stress-responsiveness (e.g. circulating levels of catecholamines and corticosteroids after a stress event) have been found to correlate with levels of activity in free swimming fish [151], and so may also be involved in shaping patterns of movement in the wild. Recent developments in stable isotope analysis now also permit the back-calculation of metabolic rates experienced by free swimming fish using otoliths [56], which could also provide a physiological perspective on fish movement when combined with telemetry data. Fish telemetry and biologging can also be used to empirically test assumptions and predictions of physiological frameworks of animal movement. For example, Gannon et al. [90] tested predictions of performance limited biogeography of dusky flathead (*Platycephalus fuscus*) by recording patterns of fish activity alongside environmental temperature via acoustic accelerometry. Flathead activity data approximated a thermal performance curve, and extrapolated estimates of zero activity were found to correspond to minimal and maximal water temperatures experienced by flathead at their latitudinal extremes. Examining fish tracking data in the context of physiological mechanisms in this way can therefore be a powerful approach to incorporating physiological context into telemetry studies where practical constraints prevent collection of physiological data on focal fish species.

### What are the major drivers of long-distance movements?

Potamadromous fishes migrate between natal and feeding areas entirely within freshwater and, although relatively shorter, these movements may be just as important for survival, growth, and reproduction as the migrations of oceanadromous or diadromous species [281]. Regardless of the type and size of the waterbody, there are several known internal (sex, life stage, size, condition, species) and external factors (water level, flow, temperature, salinity, dissolved oxygen) known to drive the long-distance movements in fish [200, 281]. The internal drivers are measurable in fish across all systems, however, the environmental factors are much more difficult to measure in a large open-water system. For example, water temperature is repeatedly identified as a driver of movement, often to exploit some form of patchy food resource or to obtain a bioenergetic advantage [82, 228]. For oceanic migrations, sea surface temperature obtained from satellites and in-situ buoys are often correlated with animal movement [204], however, this provides very little information about the subsurface temperatures that the fish are experiencing.

Telemetry studies on these lentic inland systems are common. Given their scale (relative to open ocean systems), scientists are often able to track large numbers of fish from one end of the waterbody to the other. Moreover, because water flows downstream, most lakes have various tributaries or are otherwise interconnected, providing opportunities to study migrations of various forms that are not unlike the types of migrations observed in open ocean systems or between oceans, estuaries and inland systems. Because inland systems are smaller, it is often possible to obtain much more information on environmental drivers of movements. In some cases, it may even be possible to recapture fish several times, which allows one to assess how aspects of organismal physiology relate to space use and movement. Several common themes have emerged from studies of freshwater long-distance movements that have relevance to understanding similar phenomena at ocean scales. For example, Raby et al. [237] used whole-lake telemetry in the Lake Erie to identify that while movements were directly related to behavioural thermoregulation, walleye that travelled ~ 100 km further east experienced similar temperatures and therefore, potential foraging opportunities could have driven the longer migration distances. Some movements, especially those related to reproduction or overwintering, are often triggered by a very specific water temperature. For example, a study of maturing adult sockeye salmon revealed specific temperatures when fish moved onto spawning grounds [203]. Yet, the cues can be much more diverse. Given dramatic seasonality in freshwater systems, changes in daylight with seasons can influence fish movements [17]. In more fluvial systems, flows can trigger movements and are often responsible for enticing fish to move upstream from lakes into tributaries to reproduce (e.g., [116]). Tides are not relevant in freshwater systems but it is not uncommon to study water drawdowns on fish movement in lakes/reservoirs (e.g., [29]) or fish responses to seiche events (Jill Brooks, Unpublished Data), which could both be relevant to understanding fish movements in marine systems. In some cases, fish in freshwater lakes have been observed to undertake diel bank migrations (e.g., [70]) not unlike what would be observed in marine systems during intense tides. Endocrine and other physiological triggers for migration have been studied in a number of freshwater systems. For example, Shaw et al. [268] tagged lake sturgeon with telemetry transmitters and collected blood samples to assess reproductive status. That analysis enabled the researchers to establish the endocrine characteristics associated with different levels of movement.

Environmental conditions that may drive long-distance movements of aquatic animals are difficult to measure at fine spatial resolution. Aquatic loggers are often placed in grids or lines, ideally in three dimensions to sample profiles of temperature, salinity, oxygen, light penetration, water density, current, and other hydrological parameters that fluctuate spatially and temporally in a system. Habitat mapping may be efficient to understand how substrate, depth, and flow in an area dictate the presence/absence of animals. However, the temporal extent of these measurements will dictate their efficiency. Macrophyte cover will vary seasonally and changes in water chemistry can change on short time scales, especially where point-source pollutants are present. Investigators using telemetry should be aware of how changes in water temperature, density, salinity, gas saturation, plant biomass, and other factors will affect the detection probability and the ability to confidently resolve presence or absence of animals at a given location at a specific time. Biological properties such as plankton or fish larvae can be sampled by hydroacoustic monitors, which can provide context about prey biomass in an area to be linked to the arrival/departure of fish based on detections on acoustic arrays. For long-distance migrations of freshwater fish, it has been shown that grid arrays are more efficient and statistically robust than line arrays for tracking seasonal movements and yielding data that are useful for testing hypotheses about long-distance movements [155].

### How does predation risk influence movement strategies?

From an evolutionary perspective, individual fish should strive to maximize their fitness. This implies acquiring resources (energy, material) needed for growth, maturation, and reproduction, but also surviving until successful reproduction (or in some cases helping kin survive reproduction; e.g. killer whale, *Orcinus orca*). The optimal foraging theory (OFT) predicts that animals should maximize their energy intake rate [233]. OFT may help explain prey choice, when to leave a foraging patch, etc., but it does not account for risk effects. Typically, there is a positive correlation between foraging modes and predation risk. Aiming for foraging success often involves a lower survival probability, whereas aiming for a high survival probability (in terms of avoiding predation) is associated with a reduction in foraging potential. Animals tend to behave according to certain strategies, where foraging gain to a smaller or larger degree is traded off for reduced predation risk. Because predation risk influencing foraging potentials change with body size for a growing individual, the optimal strategy may be stage-dependent and change with ontogenetic development or during periods of starvation [169]. Resolving these strategies is key to understanding ecology, life-history tactics, and evolution. In broad terms, fish can manage predation risk in multiple ways, including 1) reducing activity or visits to risky habitats, 2) seeking shelter among conspecifics (i.e., shoaling, schooling), 3) seeking shelter among structures (macrophytes, stones etc.), and 4) seeking shelter in the darkness of the deeper waters. Minimizing the ratio between predation risk and foraging gain (μ/g) is one well-known example of such strategies, first described for the optimal habitat choice of bluegill sunfish (*Lepomis macrochirus*) and for optimal size for metamorphosis in tadpoles [304, 305]. Later, minimizing μ/g was used to explain diel vertical migration in sockeye salmon the antipredator window hypothesis [57, 254]; as well as other planktivorous fish and zooplankton (e.g. [98]), where planktivorous fish feed in the plankton-rich epilimnion from dusk till dawn when predation risk from visual predators is low. Synchronization among individuals can provide protection through predator swamping, i.e. predation risk is reduced because the predator is full, or the predator gets confused by the synchronised behaviour of schooling individuals (e.g. [18]). Moreover, parasite infection may have profound effects on the behaviour of an individual, altering its susceptibility to predation [18].

Altered predation pressure, either through changes in native predator densities, or through introductions of novel predators, may have direct population effects on prey fish through consumption, but the non-consumptive effects mediated through altered behaviour of prey may be as strong [77, 152, 168] and may also lead to trait-mediated effects on other species [223]. Understanding these interactions is important from a management point of view. As an example, fish density in lakes is typically sampled as catch per unit of effort in fishing nets, but is a reduction in catches caused by truly reduced densities, by different activity or habitat use related to temperature, or by reduced activity levels or changed habitat use as a response to increased predation risk? Moreover, predator-induced changes in behaviour may also change life history tactics, and ultimately lead to evolutionary changes such as speciation or hybridization.

Lakes are ideal ecosystems to study the arms race driving predator and prey movement strategies. Regardless of size, lakes are defined by physical boundaries and especially smaller lakes can be considered as relatively closed systems. Small and moderate-sized lakes (up to a few km^2^ area) may be fully covered by acoustic tracking systems and, if instrumented with acoustic hydrophone arrays, act as upscaled field versions of video surveyed laboratory aquariums (Fig. 2). Small to medium-sized experimental ponds may also be controlled and manipulated, and much easier replicated than lakes. By tracking both predator and prey species in such systems, experimental ponds can facilitate experimentally designed single or multi-species fish communities (including multiple trophic levels) in a replicate manner, preferably with a before-after control-impact (BACI) design when possible. Examples include introduction of predators (native, neonative or invasive) to otherwise predator-void systems, removal of predators, effects of chemical and visual cues of predators, effect of habitat complexity. Through such designed experiments, underlying mechanisms of the intricate interactions between predator and prey behaviour can be studied, tested, and established. Furthermore, combining such experiments with other manipulations such as contamination by anthropogenic compounds or inoculation with pathogens should make it possible to reveal interactions. This is an important field of research, considering that there is little knowledge for aquatic animals, whereas the pressure from local, regional and global environmental contaminants and pathogens is to a large extent ever increasing.

### What areas can be considered hotspots for multiple species on a global scale?

Freshwater ecosystems represent biodiversity and conservation hotspots [276]. Biodiversity hotspots represent assemblages of multiple species and therefore require investigation of representatives of multiple trophic scales. Fishes in lake ecosystems represent suitable study objects in this respect, because they span the whole trophic scale, from primary consumers to top predators. Studies focused on freshwater fish diversity, community structure, and distribution rely mostly on lethal or harmful sampling methods, such as fishing nets, traps and electrofishing [157, 217]. However, these methods are inadequate to investigate whole fish community dynamics at a finer scale. While this led to proposals for the use of combined multiple sampling methods to better describe the fish community in lakes and reservoirs [156], such approaches are costly and still not able to capture the dynamics of fish populations at a high spatio-temporal resolution. The integration of novel tools with traditional methods seems to be the best approach to understand the fish community dynamics [65, 66] and the hotspots for lake biodiversity on a global level. While the global pattern of biodiversity hotspots is that the most diverse communities are found at lower latitudes, lakes are unevenly distributed, with higher lake density at higher latitudes [296]. Consequently, lakes do not necessarily follow the same biodiversity richness patterns as other aquatic environments. As a result, distribution patterns of fishes in lakes provides a unique perspective to understand the relationship between animal distribution patterns and latitudinal patterns in global biodiversity distribution.

Acoustic telemetry can provide insight in fish community distribution by identifying the most frequently used areas of the lake through time by different fish species (Fig. 2). One of the advantages of telemetry over traditional sampling methods is the possibility to operate well in less accessible terrains, such as shallow zones and deep or highly structured areas of lakes. Telemetry combined with environmental monitoring can provide detailed information on habitat preferences of different fish species, especially on critical habitats such as spawning, feeding, and nursery grounds, which tend to be species-specific and temporally dynamic (e.g. [26, 81, 105]). Such data allows the development of detailed maps of critical habitats for multiple species through time in a given lake [275], generating tools to improve the environmental management, such as the selection of areas within lakes that have specific characteristics that favor only target species, while inhibiting the proliferation of invasive species [184, 247]. Due to the bounded nature of lakes, understanding the habitat preference, dynamics, and activity of different fish species is easier to accomplish than in open ecosystems such as the marine environment where animals are able to move beyond monitoring areas. Therefore, the knowledge gained in lakes can contribute to better understanding of species distribution and critical areas, which can be transposed to other types of ecosystems.

One challenge when applying acoustic telemetry to assess distribution and habitat use of the whole fish community in an ecosystem is that it requires simultaneous tracking of all, or at least most, of the community members. Tracking multiple species is, however, costly, whereas the use of telemetry for some species is not possible due to small body size for transmitter implantation. In this respect, the advantage of lake ecosystems is that they can have a relatively small number of species relative to marine habitats, which makes it more affordable to conduct such studies in lakes. Furthermore, one potential way to resolve these obstacles would be to apply surrogate species concepts to fish telemetry, especially indicator and umbrella species concepts [49]. Indicator species represent organisms whose characteristics can be used as an indication of particular ecosystem aspects that are too difficult to measure for other species, or by other methods [49, 130]. The two most relevant types of indicator species in this respect are indicators of environmental health and biodiversity richness [49, 224]. Umbrella species are characterized by similar resource and habitat requirements as sympatric species, which makes management and protection measures directed at such species at the same time effective and supportive for other species [48]. Umbrella species concept has been applied in practice with mixed success, which mainly depends on the criteria used for umbrella species selection [32, 260]. In this respect, whole-lake acoustic telemetry represents a suitable approach to test and develop this concept further. If applied properly, indicator and umbrella species concepts provide an opportunity to obtain valuable information by tracking movement of just a selected set of species. Due to considerably smaller diversity of fish assemblages in lakes than in fluvial and marine systems, the surrogate species concept is likely to be comparatively more efficient in lakes, being thus a feasible alternative to gather information on the community at substantially lower costs than in other aquatic environments.

### How do anthropogenic activities (e.g., shipping, fishing, and water management) affect movements?

The impact of human activities such as fishing, stocking, shipping, construction, extraction processes and pollution on fish is a major topic addressed by a wide range of scientific disciplines, including ecology, eco-toxicology and conservation. Population-level parameters such as recruitment, abundance and size-structure have often been regarded as ultimate endpoints in many impact studies [6], however attention has increasingly been directed toward individual-level sub-lethal parameters such as behavior and movement [71]. Behavioural alterations following anthropogenic activities may potentially have severe long-term ecological effects, and lead to population decline and even extinction [286]. Until recently, studies evaluating impacts on behavior and movement have relied on traditional tag-recapture methods or low-resolution telemetry to collect data [65, 66]. These methods often generate very sparse data, with wide temporal gaps between observations, which limits the ability to evaluate impact at any detail. Moreover, technical challenges with tracking fish behavior across large spatial scales in the field have made laboratory studies the preferred method in many disciplines, e.g. ecotoxicology. Here, behavioural effects seen in the lab are extrapolated to the field, often with little or no validation of the transferability of such effects between the one-dimensional laboratory environment and the often vastly complex natural systems [119].

The ability of acoustic telemetry to track fish with high spatial and temporal resolution across large areas promise exciting new opportunities to study the impact of anthropogenic activities on fish behavior. Lakes provide the perfect platform for such studies, because they have natural boundaries that contain the fish within the receiver/tracking array, in contrast to open systems such as oceans or rivers. Dense receiver networks can be used to fine-scale position fish over the entire lake for many months or years (Fig. 2). This effectively closes the spatiotemporal gaps limiting earlier impact studies, and allows for very detailed investigations of behavioral effects at an unprecedented scale. Although still in its infancy, fine-scale tracking studies in lakes have already contributed significantly to our understanding of the behavioural impact of anthropogenic activities, such as recreational fishery, habitat degradation and damming [23, 39, 139].

A clear example of how lake-studies in combination with fine-scale acoustic telemetry have advanced the way anthropogenic effects are evaluated is its use in field ecotoxicology. Chemical pollution has long been recognized as a major threat to aquatic ecosystems worldwide [94]. Many chemicals enter waterways via treated wastewater effluent [42, 153], or from production industries [84, 251], and remain bioactive after they reach aquatic systems [76, 99]. Whereas much of the work on contaminants has focused on lethal effects associated with high chemical concentrations, dilute concentrations can alter a diverse array of behaviors, including predator–prey interactions, mating and social behaviors [60, 85, 163, 243, 317]. Until now, studying behavioral effects of chemicals have been restricted to laboratory environments, and to what extent results generated by standardized lab experiments with low complexity can be translated to the real world has been debated [148, 249]. However, due to technological advances of acoustic telemetry we now have the tools to test and validate lab-based ecological risk assessment of chemical pollution in full lake studies [119]. The implementation of acoustic telemetry in risk-assessment using lakes as study units would have multiple benefits. First, it allows for testing if chemicals identified to invoke behavioral effects in the lab also do so in the real world (e.g. [148]). Second, it allows for the introduction of ecological endpoints that we are unable to study in the lab, e.g. home-range size and habitat choice. Third, large-scale studies also allow for realistic assessments of effects on interspecific interactions such as predation and competition, and how these affect life-history characters like growth, development and ultimately mortality. The monitoring of anthropogenic effects on behavioral traits using acoustic telemetry in lakes is a much needed innovation that will increase the ecological relevance, reliability and precision of risk-assessment of different types of aquatic pollution (e.g. chemical, light, sound).

## Synthesis

Hays et al. [117] outlined questions specifically focused on marine megafaunal movement. We have interpreted them as canonical questions in movement ecology, where significant gains can be made by understanding processes and mechanisms that provide natural microcosms for biotic interactions. Although we propose study designs that could advance our understanding of fundamental principles of aquatic animal movement, these will of course be enhanced by having broad-scale studies of wide-ranging marine and terrestrial species as well. For example, studying major drivers of long-distance movements is an incredibly important frontier in improving our understanding of animal movement principles, and although partial answers can be ascertained from lakes, this question is inherently best suited to be studied in vast, unconstrained environments such as seas. Animals in lakes are range restricted and therefore it is possible to instrument entire basins. This has been demonstrated even across large areas such as Lake Erie, where a whole-lake telemetry array has been established to track fish movements basin-wide.

Logging tags can be recovered by recapturing the tagged animal or by having a pop-off mechanism on the tag package that makes it recoverable. Raby et al. [238] used such a pop-off package with depth and temperature sensors on Great Lakes salmonids to compare the niche of native lake trout (*Salvelinus namaycush*) and introduced chinook salmon (*Oncorhynchus tschychawa*). Lack of two-dimensional (latitude-longitude) data from this method, however, was identified as a limitation by the authors. Positioning of aquatic animals can be accomplished by Fastloc GPS tags that connect to satellites and relay information when animals break the surface [80], by calculating Doppler shift from Argos satellites, or by light-based geolocators (see [107]). However, positioning of tags in space across small areas can be accomplished by acoustic telemetry. Transmitters fitted with pressure sensors can then be used to model three-dimensional space use using kernel smoothing methods, hidden Markov models, or network analysis to investigate the spatial distribution of the animals within environments such as lakes where there is sufficient spatial coverage of receivers to generate reliable animal presence data.

Whole-lake studies are challenged by factors affecting detection distance and the probability that a tagged animal will be reliably detected. Environmental conditions can alter the detection radius of a receiver and synchronization tags or reliable range testing are necessary to accurately position animals. Marine environments are characterized by similar challenges in the form of boat noise, tidal streams and maelstroms, as well as biological noise from species such as shrimp. The largest lakes can encounter substantial boat noise but smaller lakes will be exposed to smaller crafts and slower motors. Hydropower facilities, however, can greatly influence the noise routine in a lake, and affect the detection distance of receivers, which may in turn influence position estimation. Phenomena such as gas supersaturation may also affect acoustic receiver detection radius.

Many ecological phenomena are manifested at particular scales, which have to be matched by an appropriate study system scale to properly address a movement ecology question (Fig. 1). Ponds and small lakes, where individuals can easily access the entire habitat and differentiated niches exist for only a small number of species, can easily be replicated and used as experimental units in studies requiring better control. Investigating mechanisms of fish movement may be optimal in relatively small natural systems where many individuals can be tracked at higher spatiotemporal resolution (Fig. 2) than at larger systems, with fewer confounding factors, higher capacity to quantify the dynamics of relevant environmental factors, and greater potential for replications in a more experimental design. For example, this can include studies of responses to anthropogenic activities and predation risk, sensory information used for navigation, and the role of social interactions. Larger systems with more habitat heterogeneity and biodiversity are ideal systems for addressing questions requiring ecological realism. For example, how climate change could impact fish movements, what habitat features create ecological hotspots, and drivers of complex long-distance movements require large, heterogeneous habitats to investigate fish movement. Larger systems may be more challenging to replicate because of the smaller degree of control that can be exerted on these systems and the greater expense associated with such large-scale monitoring. Ultimately, the scale of the study will dictate the results; larger, replicated, controlled studies will reveal more than observational studies in smaller, unreplicated, and uncontrolled ones, but only if they are conducted robustly with good coverage and consideration of potential pitfalls (e.g. fish emigration, unreliable receiver detections, poor characterization of environmental dynamics, or cryptic predation). Recent progress in terrestrial wildlife tracking has pushed forward the spatial and temporal trade-offs that have long limited movement ecology research, enabling high-resolution data on wild animals and their natural environment across much larger areas, longer durations and many more interacting individuals/species compared to traditional tracking systems [285]. Acoustic telemetry provides the means to push forward such limits in aquatic and coastal ecosystems as well.

Lakes are seminal habitats for studying ecological theory and research in these habitats has been a catalyst for revealing important paradigms relevant to trophic ecology (e.g. [244, 310]) and relationships between animals and their habitat [239]. We have submitted here ways in which aquatic telemetry systems deployed in lakes now have the potential to play a key role in unravelling many of the remaining questions in movement ecology, in particular when combined with environmental data with high spatiotemporal resolution [117]. Necessarily, these systems will predominantly use fish as model species, but there are exciting opportunities for other species. Although aquatic invertebrates are more abundant and diverse than fish, fish are more readily tagged than insect larvae given their size and use more of the water column. Moreover, many insects are aquatic only as nymphs and their adult phases emerge to become terrestrial. Larger invertebrates such as crayfish and *Bellostomata* spp. can carry tags and may be integrated into some whole-lake studies and there is a promising future for tracking aquatic insects with miniaturization of tags [74]. Predatory birds, particularly cormorants, herons, kingfishers, and ducks, along with mammals such as mink and otter, as well as turtles and crocodilians, can spend significant time in the water and could also be tagged to study predator landscapes, but will require specific tagging protocols adapted to meet the unique welfare needs of these animals. Ichthyofauna will nevertheless be the preferred study system for fundamental ecological questions, either studying a single sentinel species, a pair of interacting species, or several members of a fish community. These studies will benefit from understanding the abundance of the tagged species and the proportion of the population carrying tags, and therefore also the demographic rates of the tagged species to ascertain how quickly this proportion will decline as tagged and untagged fish die, and untagged fish are born into the population. Individual studies may investigate questions in single lakes and later become integrated within meta-studies to answer new questions as part of post-hoc replicated experiments. For applied research, there is a growing need to forecast movement behaviour, e.g. to understand vulnerability to angling [194] or responses to potential mitigation measures, and simulation studies parameterized by tracking studies of wild fish have a great potential to this end [278].

Mining the benefits of whole lake telemetry for conservation and management depends on a number of challenges. Besides the obvious obstacles of resources to equip entire lakes with hydrophones and manage the big data stream, key issues revolve around the difficulty of obtaining random fish samples as all gears suffer from gear biases in terms of which behavioural types are captured for tagging [312]. Moreover, tag life can limit the observation time window and may not allow us to arrive at assessments of whole life-cycles and ecosystem responses to experimental perturbations. Tagging effects can affect growth and fecundity and thereby limit how well the tagged fish represent the fish population. As pointed out earlier in this paper, there is a particular need for more research on small fish, and development of smaller acoustic tags is needed. Clearly, some of the experiments proposed above may also be ethically problematic, in particular when destructive experiments (e.g., release of pollutants) are conducted. Nonetheless, there are a growing number of experimental lake facilities where such work can be conducted (e.g., Experimental Lakes Area in northwestern Ontario, Canada), and new environmentally safe methods for exposing fish to chemicals in the field using implants is currently being developed [180].

Work on threatened species might also be limited as tagging demands invasive procedures that may not be welcomed by authorities and the public at large. Alternatively when pairing telemetry with other novel ecological methods (e.g., eDNA, stable isotopes, genetic tagging, genomics, ecoacoustics) possibly entire lakes can be transformed into large, real-life laboratories to tackle pertinent questions of management, and assessment and conservation at the right ecological and managerial scales. Clearly, lakes could offer test arenas to tackle questions that will not be possible to address in more open systems such as the ocean. Whereas marine fisheries has a tendency to be largely based on modelling, freshwater ecology tends to emphasize ecological questions in fish ecology. By combining modelling techniques for population dynamics developed in the marine realm with the functionality of being able to measure detailed ecological processes (e.g., exchange dynamics among refuge and open habitat [178]) in the freshwater ecosystem, a revolution in fish and fisheries sciences with relevance to management can be aspired to from whole-lake studies involving telemetry.

Networks for archiving animal movement data can facilitate whole-lake telmetry studies so that data are available to contemporary researchers and future generations [212]. The Ocean Tracking Network and European Tracking Network can act as hubs for connecting researchers with relevant data, and ensuring that these data are accessible for meta-studies that can integrate research from multiple lakes to ask new questions. In order for this to be possible, researchers must be committed to collecting and archiving metadata about their study system, including technical details such as receiver detection radius and especially environmental parameters such as temperature, lake stratification, and species assemblage.

## Data Availability

No data were generated for this manuscript and none are available for further use.
