# Correction to: A role for lakes in revealing the nature of animal movement using high dimensional telemetry systems

**DOI:** 10.1186/s40462-021-00285-3

**Published:** 2021-10-20

**Authors:** Robert J. Lennox, Samuel Westrelin, Allan T. Souza, Marek Šmejkal, Milan Říha, Marie Prchalová, Ran Nathan, Barbara Koeck, Shaun Killen, Ivan Jarić, Karl Gjelland, Jack Hollins, Gustav Hellstrom, Henry Hansen, Steven J. Cooke, David Boukal, Jill L. Brooks, Tomas Brodin, Henrik Baktoft, Timo Adam, Robert Arlinghaus

**Affiliations:** 1 Laboratory for Freshwater Ecology and Inland Fisheries (LFI) at NORCE Norwegian Research Centre, Nygårdsporten 112, 5008 Bergen, Norway; 2 INRAE, Aix Marseille UnivPôle R&D ECLA, RECOVER, 3275 Route de Cézanne - CS 40061, 13182 Cedex 5 Aix-en-Provence, France; 3 Institute of Hydrobiology, Biology Centre of the Czech Academy of Sciences, České Budějovice, Czech Republic; 4 Movement Ecology Lab, Department of Ecology, Evolution, and Behavior, Alexander Silberman Institute of Life Sciences, The Hebrew University of Jerusalem, 102 Berman Bldg, Edmond J. Safra Campus at Givat Ram, 91904 Jerusalem, Israel; 5 Institute of Biodiversity, Animal Health and Comparative Medicine, College of Medical, Veterinary and Life Sciences, University of Glasgow, Graham Kerr Building, Glasgow, G12 8QQ UK; 6 Faculty of Science, Department of Ecosystem Biology, University of South Bohemia, České Budějovice, Czech Republic; 7 Norwegian Institute of Nature Research, Tromsø, Norway; 8 University of Windsor, Windsor, ON Canada; 9 Department of Wildlife, Fish, and Environmental Studies, Swedish University of Agricultural Sciences, Umeå, Sweden; 10 Karlstads University, Universitetsgatan 2, 651 88 Karlstad, Sweden; 11 Fish Ecology and Conservation Physiology Laboratory, Department of Biology, Carleton University, Ottawa, ON Canada; 12 Institute of Entomology, Biology Centre of the Czech Academy of Sciences, České Budějovice, Czech Republic; 13 Technical University of Denmark, Vejlsøvej 39, Building Silkeborg-039, 8600 Silkeborg, Denmark; 14 Bielefeld University, Universitätsstraße 25, 33615 Bielefeld, Germany; 15 Department of Biology and Ecology of Fishes, Leibniz Institute of Freshwater Ecology and Inland Fisheries, Bergen, Germany; 16 Division of Integrative Fisheries Management, Humboldt-Universität Zu Berlin, Bergen, Germany

## Correction to: Movement Ecology (2021) 9:40 10.1186/s40462-021-00244-y

Following publication of the original article [1], the authors identified errors in the affiliation assignment. For Robert Arlinghaus, the correct affiliation assignment is 15 and 16, instead of 10, 15 and 16. For Henry Hansen, the correct affiliation assignement is 10 and 15, instead of 10.

The affiliation list has been updated above and the original article [1] has been corrected.
